# Dre-miR-2188 Targets *Nrp2a* and Mediates Proper Intersegmental Vessel Development in Zebrafish Embryos

**DOI:** 10.1371/journal.pone.0039417

**Published:** 2012-06-22

**Authors:** Ana R. Soares, Marisa Reverendo, Patrícia M. Pereira, Olivier Nivelles, Hélène Pendeville, Ana Rita Bezerra, Gabriela R. Moura, Ingrid Struman, Manuel A. S. Santos

**Affiliations:** 1 RNA Biology Laboratory, Department of Biology & CESAM, University of Aveiro, Aveiro, Portugal; 2 Unit of Molecular Biology and Genetic Engineering, GIGA-Research, University of Liège, Sart Tilman, Liège, Belgium; IRCCS-Policlinico San Donato, Italy

## Abstract

**Background:**

MicroRNAs (miRNAs) are a class of small RNAs that are implicated in the control of eukaryotic gene expression by binding to the 3′UTR of target mRNAs. Several algorithms have been developed for miRNA target prediction however, experimental validation is still essential for the correct identification of miRNA targets. We have recently predicted that Neuropilin2a (*Nrp2a*), a vascular endothelial growth factor receptor which is essential for normal developmental angiogenesis in zebrafish, is a dre-miR-2188 target.

**Methodology:**

Here we show that dre-miR-2188 targets the 3′-untranslated region (3′UTR) of *Nrp2a* mRNA and is implicated in proper intersegmental vessel development *in vivo*. Over expression of miR-2188 in zebrafish embryos down regulates *Nrp2a* expression and results in intersegmental vessel disruption, while its silencing increases *Nrp2a* expression and intersegmental vessel sprouting. An *in vivo* GFP sensor assay based on a fusion between the GFP coding region and the *Nrp2a* 3′UTR confirms that miR-2188 binds to the 3′UTR of *Nrp2a* and inhibits protein translation.

**Conclusions:**

We demonstrate that miR-2188 targets *Nrp2a* and affects intersegmental vessel development in zebrafish embryos.

## Introduction

MicroRNAs (miRNAs) regulate gene expression at the translational level by binding to the 3′untranslated region (3′UTR) of target mRNAs and by activating their degradation or inhibiting their translation [1]–[4]. Eukaryotic genomes encode hundreds to thousands of miRNAs [2] and a single miRNA can regulate the expression of hundreds of genes while one gene can be regulated by more than one miRNA [1], [5], [6]. These molecules are implicated in the regulation of several biological processes, are important for normal development and physiology [7]–[11] and their deregulation is implicated in disease [12]–[14]. Knowledge of miRNA targets is therefore pivotal to understand the function of these small non-coding RNA molecules. Our group has recently identified novel zebrafish miRNAs and identified putative targets for some of those miRNAs [15]. Our algorithm predicted *Nrp2a*, a VEGF co-receptor involved in developmental angiogenesis, as a miR-2188 target suggesting that this miRNA may be involved in the regulation of this process.

Neuropilins (Nrps) are expressed in the vascular system with some degree of vessel type specificity in human, mouse, chicken and zebrafish embryos [16] and mediate normal developmental angiogenesis [17]–[19], which is a process by which new blood vessels form in response to external stimuli sensed by vascular endothelial cells. Disruption of this process leads to abnormal vessel growth and contributes to ischemic, inflammatory and immune disorders [20]. Although Nrps are not able to initiate signaling pathways by themselves and require interaction with other co-receptors, such as the VEGFRs (VEGFR-1, VEGFR-2 and VEGFR-3) [21], their deregulation is implicated in pathological angiogenesis in tumors and retinal disease [22], [23].

In zebrafish there are 4 Neuropilin genes, namely *Nrp1a*, *Nrp1b*, *Nrp2a* and *Nrp2b* [19], [24]. *Nrp2a*, the predicted target of miR-2188, is an ortholog of human *Nrp2* that interacts with VEGFR2 and is required for correct development of major intersegmental vessels (ISVs) in zebrafish embryos [19], [24]. Knocking down *Nrp2a* in zebrafish embryos results in arteriovenous malformations in the tail and irregular ISV patterning, while double knockdowns (*Nrp2a* and *Vegf*) increase vasculature defects and circulation arrest, which is not observed in single knockdowns [19]. In other words, there is physiological interdependence between *Nrp2a* and *Vegf* during embryonic development and *Nrp2a* is likely involved in angiogenesis. Indeed, over expression of *Nrp2*, the human ortholog of *Nrp2a*, enhances vessel proliferation and migration induced by VEGF, suggesting that *Nrp2* increases the response to VEGF and plays an important role in vessel proliferation [25]. However, little is known about the regulation of *Nrp2a* by miRNAs.

Several studies implicate miRNAs in the regulation of angiogenesis. The miRNAs implicated in angiogenesis can be classified as pro-angiomiRs and anti-angiomiRs. The former promotes angiogenesis by targeting negative regulators of angiogenesis and the latter inhibits angiogenesis by targeting positive regulators of angiogenesis [26]. For instance, miR-92a (miR-17∼92 cluster) is expressed in vascular endothelial cells and suppresses the function of pro-angiogenic proteins by inhibiting translation of their mRNAs [27]. In zebrafish embryos, over expression of this anti-angiomiR causes severe defects in vessel formation [27]. Conversely, miR-126 is a pro-angiomiR and promotes VEGF signaling by inhibiting the negative regulators of the VEGF pathway, i.e., *Spred1* and *Pik3r2* [28], [29]. This miRNA is required for the maintenance of vascular structure *in vivo* in mice and in zebrafish and works as an endothelial cell-specific regulator of angiogenic signaling. Deletion of miR-126 causes loss of vascular integrity which is characterized by leaky vessels, hemorrhage and partial embryonic lethality in mice [29]. In zebrafish, knockdown of this miRNA also causes hemorrhage and collapse of the dorsal aorta and primary cardinal veins, indicating that miR-126 function is conserved in vertebrates [28]. miR-296 is also a pro-angiomiR that promotes angiogenesis *in vitro* [30] and injection of a miR-296 antagomir inhibits glioma angiogenesis *in vivo*, supporting a role for this miRNA in promoting angiogenesis in tumors [26]. These findings indicate therefore that the VEGF pathway and angiogenesis can be regulated at different levels by miRNAs, suggesting that elucidation of the biology of angiomiRs is fundamental to understand angiogenesis.

Here, we show that dre-miR-2188 targets *Nrp2a* and has an important role in modulating ISV development in zebrafish embryos. Over expression of miR-2188 in Tg(flk1-GFP)s843 zebrafish embryos, in which GFP is expressed in vascular endothelial cells under the control of *Flk (VEGFR2)*, down regulates *Nrp2a*. This down regulation is accompanied by thinner and underdeveloped ISVs, without through connections with the dorsal longitudinal anastomotic vessel (DLAV). In some cases, lack of one or more ISVs is observed. We also validate *Nrp2a* as an *in vivo* target of miR-2188 demonstrating that miR-2188 modulates the expression of a positive regulator of angiogenesis. This is the first report of control of Nrp2a by a miRNA and indicates that the development of proper ISVs during development is dependent on the regulation of *Nrp2a* levels by a miR-2188 in zebrafish.

## Results

### miR-2188 Inhibits *Nrp2a* Expression

**Figure 1:**
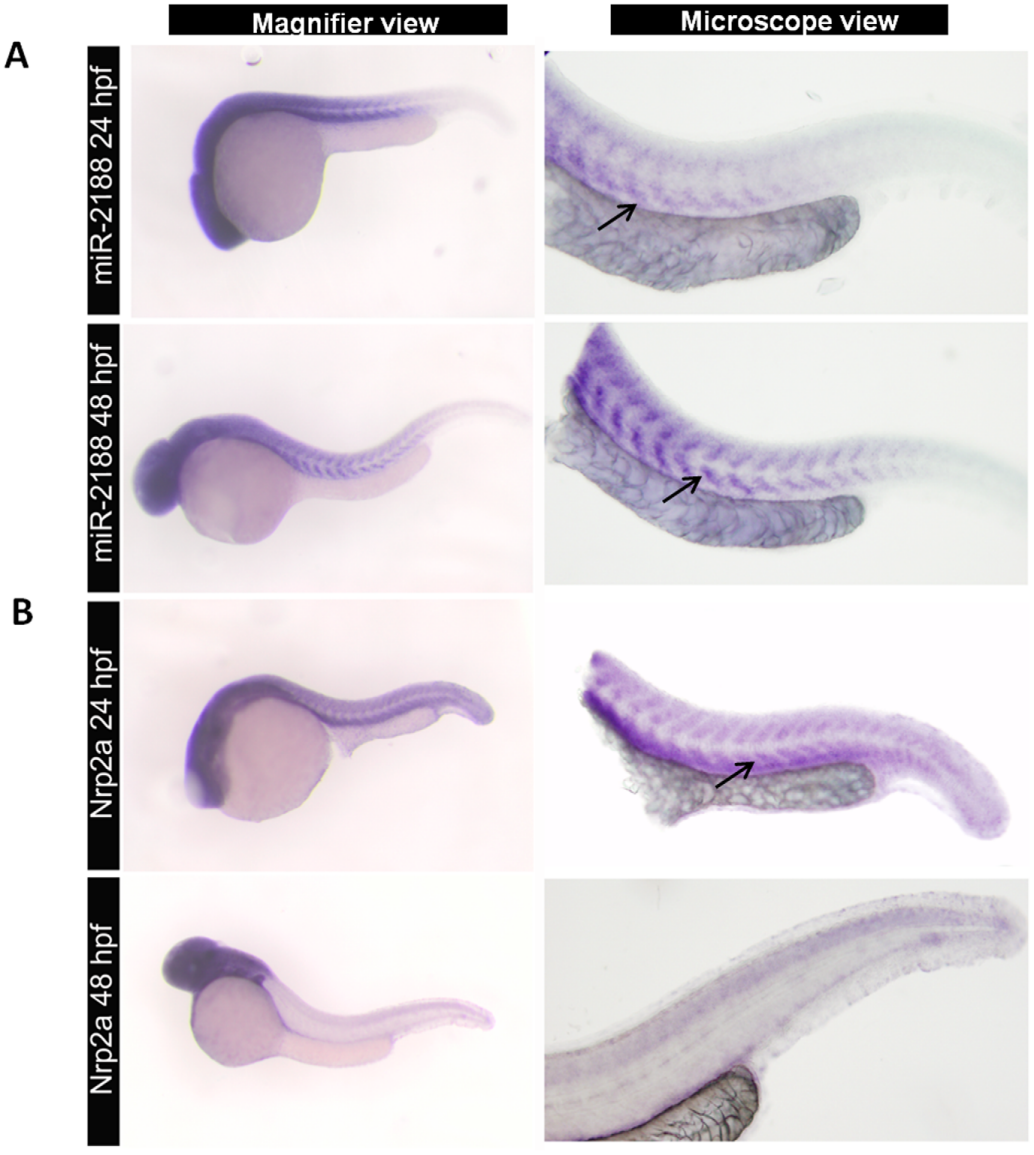
Expression of miR-2188 and *Nrp2a* in zebrafish embryos. A) ISH of miR-2188 at 24 hpf showed low expression on the trunck and tail, however it increased at 48 hpf between the somite boundaries. Arrows indicate hybridization signal. B) *Nrp2a* ISH at 24 and 48 pf. At 24hpf *Nrp2a* was predominantly expressed in the head and somitic regions, while at 48 hpf its expression was restricted to the brain and romboencephalon. There was an indirect correlation between miR-2188 and *Nrp2a* expression. Increasing miR-2188 levels through development were accompanied by decreasing *Nrp2a* expression between the somite boundaries. All embryos are in lateral view, anterior to left. Microscopic pictures were taken with 6,3x magnification.

We have identified the zebrafish miR-2188 in a previous study by deep DNA sequencing [15] and have used computational tools to predict its putative targets. This analysis showed that miR-2188 has two putative binding sites in the 3′UTR of *Nrp2a* (Table 1). In order to experimentally validate this prediction, we have analyzed miR-2188 and *Nrp2a* expression in zebrafish embryos by *in situ* hybridization (ISH). Since miRNAs regulate gene expression, a negative correlation between miRNA and target expression is generally observed. In other words, the increase of miR-2188 expression in specific tissues should be accompanied by a decrease in *Nrp2a* expression. MiR-2188 was detected at low levels at 24 hpf, but its expression increased at 48 hpf throughout the embryo tail (Figure 1-A). On the other hand, *Nrp2a* was widely expressed at 24 hpf but its expression became restricted to the brain and romboencephalon after 48 hpf, confirming previous studies (Figure 1-B) [19], [24].

**Table 1:** Putative binding sites of miR-2188 in *Nrp2a* 3′UTR.

*Nrp2a* predictedbinding sites	Match	Mfe (kcal/mol)
**Binding site-1 (BS-1)**	target 5‘ G G C U C C 3′GGA AU UG GG TGGACCUUCCU UA AC CC ACCUGGAAmiRNA 3‘ G C U A 5'	−26,9
**Binding site-2 (BS-2)**	target 5‘ U AAC A A 3′GCA GGGUU GGACCUUGU UCCAA CCUGGAmiRNA 3‘ CC ACAC A 5′	−22,1

**Figure 2:**
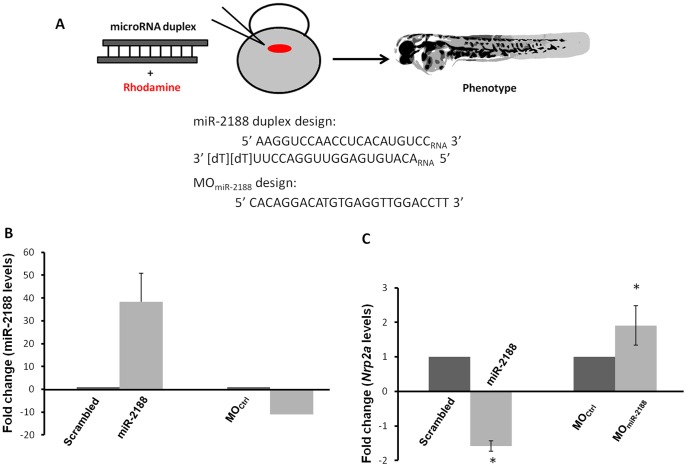
miR-2188 duplex and morpholino injections. A) A miRNA duplex and a MO_miR-2188_ were designed to over express and knockdown miR-2188 in zebrafish embryos, respectively. Duplex microinjections were performed in one cell stage embryos with rhodamine to visualize the injection process. Approximately 1000 pL of each duplex were injected. 2 µM of each duplex were chosen as the working concentration for the injections, as this duplex concentration did not induce high mortality rates or unspecific side effects. B) qPCR quantification of miR-2188 showed that miR-2188 duplex injection increased miR-2188 expression by 40 fold and MO_miR-2188_ injection decreased miR-2188 expression by 12 fold. C) qPCR quantification of *Nrp2a* levels in 48 hpf embryos after over expression and knockdown of miR-2188 showed that this target was down regulated 1.6 fold after miR-2188 over expression and was up regulated 2 fold after miR-2188 knockdown.

**Figure 3:**
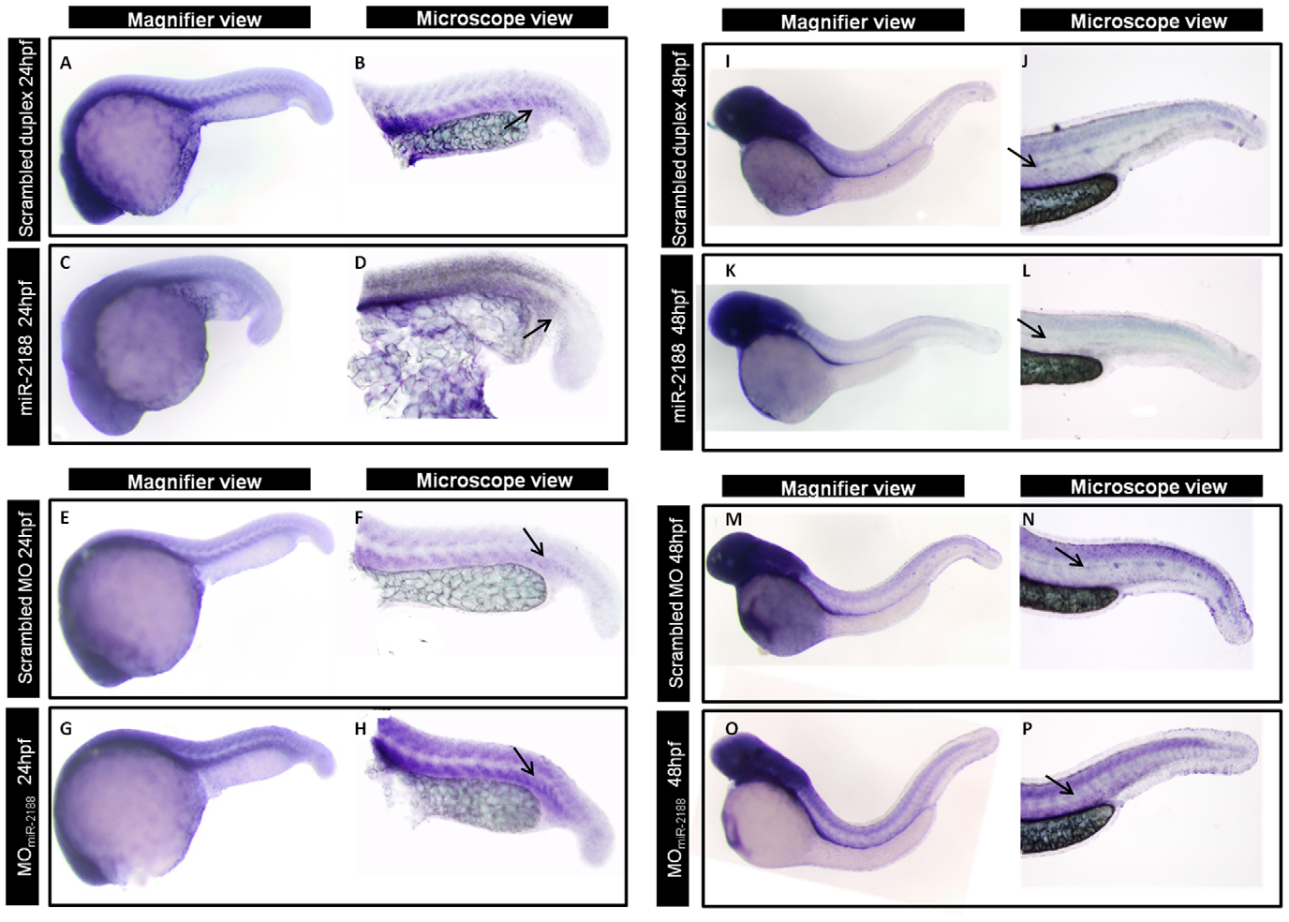
ISH of *Nrp2a* after miR-2188 deregulation. A) After injection of miR-2188 duplex, *Nrp2a* detection decreased between the somite boundaries at 24 hpf. On the other hand, in miR-2188 morphants, *Nrp2a* detection was more prounounced throughout the trunk and tail region, between the somite boundaries, when compared with the control. B) At 48 hpf and after miR-2188 overexpression, *Nrp2a* was not detected in the blood vessels or in the PCV when compared with the control embryos. On the other hand, miR-2188 morphants express *Nrp2a* in the trunck and tail, especially between the somite boundaries, which is not detected in 48 hpf control embryos. All embryos are in lateral view, anterior to left. Arrows indicate hybridization signal. Microscopic pictures were taken with a 6,3x magnification.

To test whether *Nrp2a* was a direct target of miR-2188, we have induced miR-2188 deregulation by injecting a synthetic miR-2188 duplex to over express it or a morpholino (MO) to inhibit it, in one cell zebrafish embryos (Figure 2-A). qPCR quantification showed that over expression of miR-2188 decreased expression of *Nrp2a*, whereas miR-2188 knockdown increased *Nrp2a* expression in 48 hpf embryos (Figure 2-B, C). ISH confirmed qPCR data as there was a decrease in *Nrp2a* signal upon miR-2188 over expression when compared with scrambled duplex injected embryos at both 24 hpf (Figure 3– A-D) and 48 hpf (Figure 3– I-L). Moreover, there was an increase in *Nrp2a* signal between the somite boundaries after injection of the MO_miR-2188_ at both 24 hpf (Figure 3– E- H) and 48 hpf (Figure 3– M-P)). These results are consistent with the target/anti-target theory which postulates that miRNAs and their targets are expressed in a non-overlapping manner, either spatially or temporally [31].

**Figure 4:**
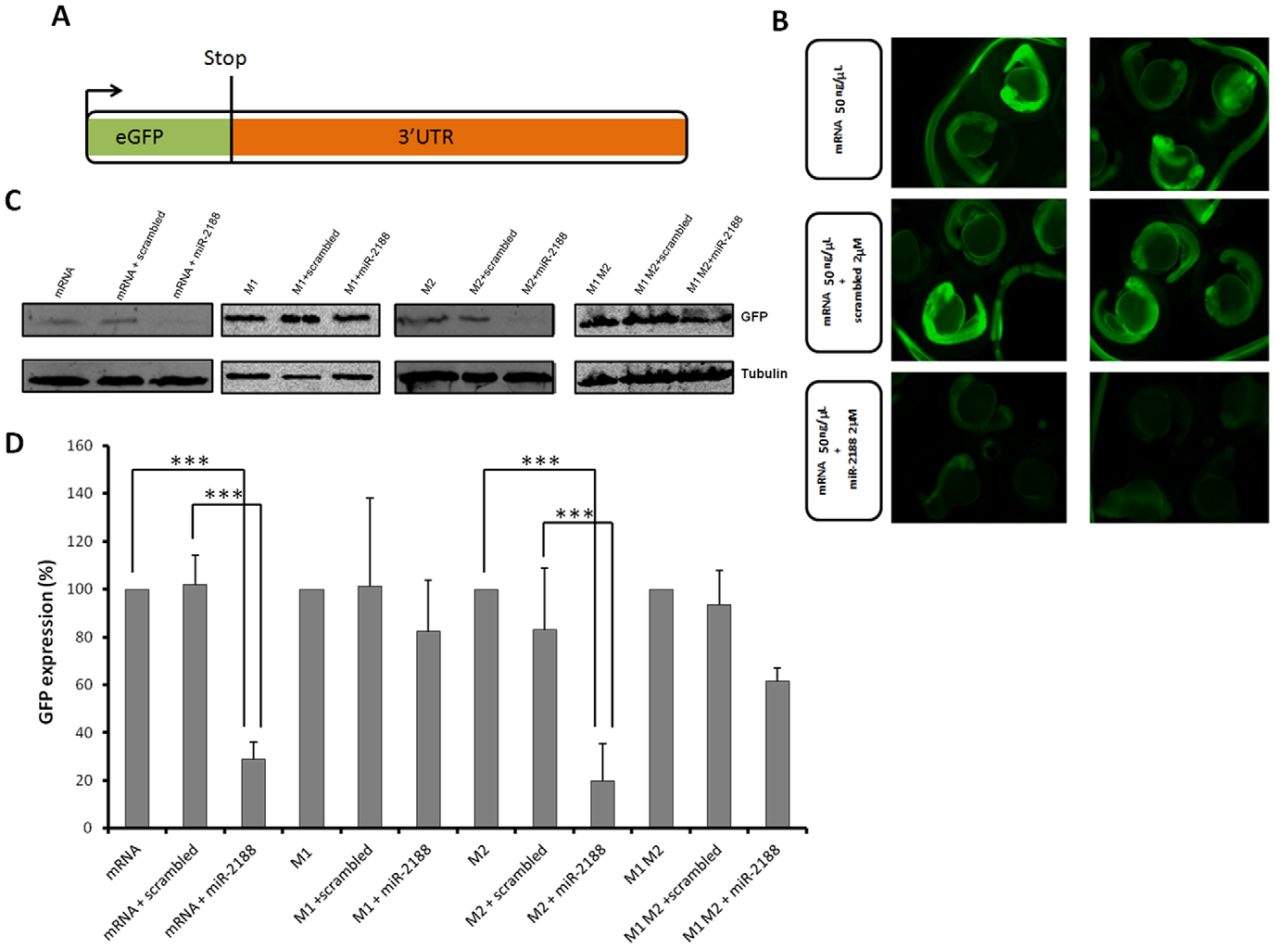
GFP sensor assay. A) The GFP gene was fused with the 3′UTR of *Nrp2a* that contained the putative binding sites of miR-2188. This construct was transcribed *in vitro* into capped mRNA prior to injection. B) Single cell embryos were injected with the GFP reporter (50 ng/µL) in the presence or absence of miR-2188 and scrambled duplexes. Fluorescence levels were observed at 24 hpf and representative embryos of each condition were photographed. Decreased fluorescence was observed in miR-2188 duplex injected embryos. C) Embryo lysates were prepared from 24 hpf embryos injected with the wild type GFP reporter or the mutated reporters in the presence or absence of miR-2188 and scrambled duplexes. Protein levels were determined by western blot analysis and β-tubulin was used as an internal control. **D)** Quantification of the reporter proteins (%) in the conditions tested using 3 biological replicates. Asterisks indicate conditions where GFP expression was significantly down regulated by miR-2188 relative to control conditions. miR-2188 injected embryos showed a statistically significant down regulation of GFP levels, indicating that *Nrp2a* 3′UTR is a true miR-2188 target. Co-injection of the M1 reporter with the miR-2188 duplex produced normal GFP levels, indicating that the BS-1 mutation abolished miR-2188 binding. On the other hand, a significant decrease in GFP expression was observed after co-injection of the M2 reporter with miR-2188, relative to the controls, indicating that BS-2 was not critical for miR-2188 binding. Data are mean +/−stdev, p<0,005 (t test, unpaired), n>3. All lanes were normalized to the β-tubulin signal.

To further test if *Nrp2a* was a true target of miR-2188, we have constructed a chimeric sensor assay based on a fusion between the GFP coding region and the 3′UTR of *Nrp2a*, which contained the putative miR-2188 binding sites or mutated miR-2188 binding sites (Figure 4-A). As expected, there was a strong down regulation of GFP levels in the miR-2188 injected embryos (Figure 4-B, C). On the other hand, binding site-1 mutation (M1), but not binding site-2 mutation (M2), in the 3′UTR of *Nrp2a* abolished the inhibitory activity of miR-2188 on GFP expression (Figure 4-C), demonstrating that *Nrp2a* is targeted *in vivo* by miR-2188 and that its down regulation is mediated by a single binding site (binding site −1) present in its 3′UTR.

**Figure 5:**
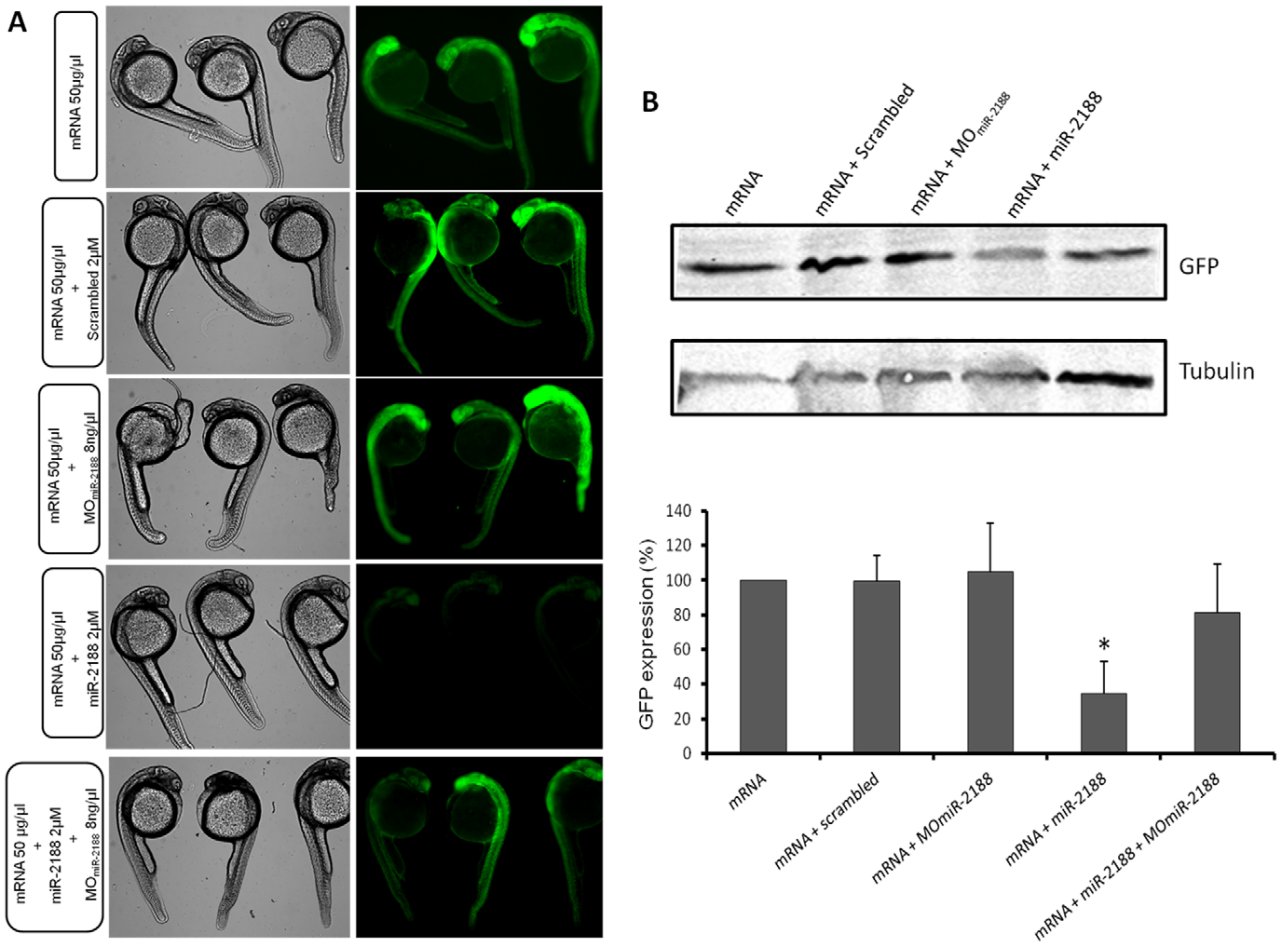
GFP rescue sensor assay. A) Single cell embryos were injected with the GFP reporter (50 ng/µL) in the presence or absence of MO_miR-2188_, miR-2188 and scrambled duplexes or a mixture of both MO_miR-2188_ and miR-2188 duplex. Fluorescence levels were observed at 24 hpf and representative embryos of each condition were photographed. Fluorescence was recovered in MO_miR-2188_+ miR-2188 injected embryos, when compared to miR-2188 duplex injected embryos. B) Embryo lysates were prepared from 24 hpf embryos injected with the GFP reporter in the presence or absence of MO_miR-2188_, miR-2188 and scrambled duplexes or a mixture of MO_miR-2188_ and miR-2188. Protein levels were determined by western blot analysis and β-tubulin was used as an internal control. **C)** Quantification of the reporter proteins (%). Asterisks indicate conditions where GFP expression was significantly down regulated by miR-2188 relative to control conditions. miR-2188 injected embryos showed a statistically significant down regulation of GFP levels, indicating that *Nrp2a* 3′UTR is a true miR-2188 target. Co-injection of the MO_miR-2188_ with the miR-2188 duplex resulted in the rescue of GFP fluorescence, further confirming that *Nrp2a* is a bona fide target of miR-2188. Data are mean +/−stdev, p<0,005 (t test, unpaired), n>3. All lanes were normalized to the β-tubulin signal.

To further confirm that *Nrp2a* is a bona fide target of miR-2188, we have performed rescue experiments where a Mo_miR-2188_ was injected in the presence of the miR-2188 duplex (Figure 5). Co-injection of the Mo_miR-2188_ and miR-2188 duplex resulted in the recovery of the GFP expression, as observed both by fluorescence microscopy (Figure 5-A) and western blotting (Figure 5-B), confirming that *Nrp2a* is a miR-2188 target. Moreover, the injection of a scrambled miRNA sequence had no effect on the fluorescence levels and there was a slight increase of GFP expression on miR-2188 morphants, as expected (Figure 5).

### miR-2188 Affects Intersegmental Vessel Development

Since miR-2188 targets *Nrp2a*, which is a VEGF co-receptor required for the correct development of major ISVs, we expected disruption of this process in miR-2188 deregulated embryos. To test this hypothesis we have used transgenic zebrafish embryos expressing GFP in vascular endothelial cells under the control of *Flk* [Tg(flk1-GFP)s843].

**Figure 6:**
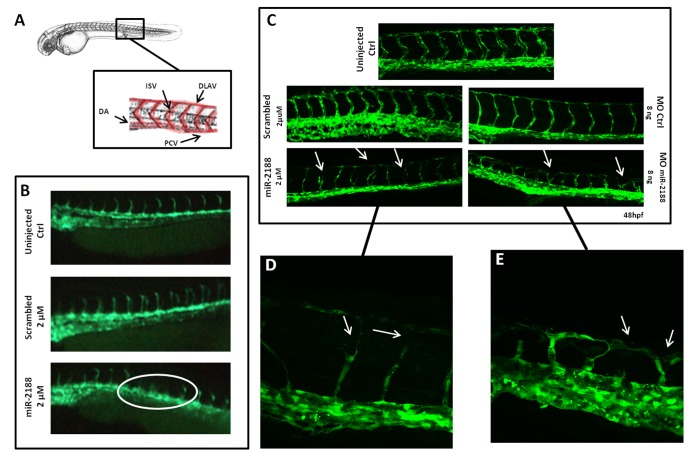
Analysis of blood vessel formation in Tg(flk1-GFP)s843 embryos. A) Representation of the zebrafish circulatory system showing the major structures (DLAV - Dorsal Longitudinal Anastomotic Vessel; ISV – Intersegmental Vessel; DA – Dorsal Aorta; PCV – Posterior Cardinal Vein). B) Visualization of 24 hpf embryo blood vessels using fluorescence microscopy. After miR-2188 duplex microinjection under developed and absent ISVs were observed, non injected and scrambled duplex injected embryos did not show such defects. C) Visualization of 48 hpf embryo blood vessels using confocal microscopy (20x). ISVs of miR-2188 injected embryos were thinner than those of non injected and scrambled duplex injected embryos and displayed ISV’s patterning defects (arrows). MO_miR-2188_ injected embryos revealed DLAV defects and branching of ISVs (arrows). Images shown in D) and E) are 60x amplification images of miR-2188 duplex and miR-2188-MO injected embryos, respectively.

Nrp2 family members interact with FLK (VEGFR2) and with other co-receptors to initiate the signaling pathways [21], thus it is expectable that alteration in FLK levels is observed in transgenic embryos after miR-2188 over expression. Previous studies have shown that knocking down *Nrp2a* resulted in arteriovenous malformations in the tail and irregular ISV patterning in zebrafish embryos [19]. A similar phenotype was induced by miR-2188 over expression (Figure 6, Figure S1). A delay or even absence of ISV formation was observed at 24 hpf, likely due to a decrease in *Nrp2a* expression caused by miR-2188 over expression (Figure 6– B). This was confirmed at 48 hpf when ISVs were already formed and differences in vessel development could be observed by confocal microscopy (Figure 6 - C-E). Control embryos (uninjected and scrambled injected embryos) had fully developed and thick ISVs whereas miR-2188 injected embryos had thinner, underdeveloped ISVs, with abrupt interruptions, lacking complete connections with the dorsal vessel (Figure 6-B-D). On the other hand, injection of the MO_miR-2188_ induced ISV branching that often resulted in DLAV defects (Figure 6-C, E). Therefore, over expression and knocking down of miR-2188 affected angiogenesis differentially; supporting the hypothesis that deregulation of miR-2188 interferes with ISV development by regulating *Nrp2a* expression.

At 48 hpf 50% of the miR-2188 injected embryos displayed circulation arrest and 70% had pericardial edema (Figure S1). Absence of one or more ISVs was observed in 60% of the miR-2188 injected embryos (Figure S1). These results indicated that miR-2188 affected angiogenesis in zebrafish embryos and were further substantiated by the injection of a MO_miR-2188_ that blocked processing and inhibited the activity of this miRNA. MO_miR-2188_ injected embryos were mainly characterized by irregular development of ISVs characterized by branching that often resulted in DLAV defects when compared to control embryos (60%; Figure S1; Figure 6– C, E). This phenotype was different from the phenotype obtained after miR-2188 over expression, indicating that over expression and knockdown of this miRNA affected angiogenesis distinctly, supporting the hypothesis that deregulation of miR-2188 interferes with angiogenesis.

This hypothesis was further confirmed by rescue experiments where MO_miR-2188_ was injected in embryos overexpressing miR-2188. Co-injection of MO_miR-2188_ with the miR-2188 duplex rescued ISV patterning, indicating that de-regulated levels of miR-2188 induce ISV defects (Figure S2).

## Discussion

Experimental validation of miRNA targets is essential to understand the functions of these small RNA molecules. Experimental validation of predicted targets is often achieved by miRNA gain or loss-of-function followed by monitoring mRNA and protein levels of the predicted targets, or by monitoring the activity of a reporter gene whose mRNA 3′UTR contains miRNA target sites [32], [33].

In this study, we have experimentally validated the miR-2188 target prediction obtained in a previous study [15]. The data show that miR-2188 regulates *Nrp2a*, a VEGF co-receptor, which is required for proper development of major ISVs in zebrafish [19]. Neuropilins increase the response to VEGF and are up regulated in endothelial tumor cells [25]. The identification of a miRNA that regulates vessel development and integrity by targeting a VEGF receptor has therefore implications for vascular development.

We have demonstrated that the *Nrp2a* 3′UTR is targeted by miR-2188 in zebrafish embryos and that this miRNA interferes with ISV development. Its over expression produced a similar phenotype to that induced by a *Nrp2a* MO, supporting the hypothesis that miR-2188 is implicated in angiogenesis by regulating *Nrp2a* expression [19]. As expected, *Nrp2a* levels are down regulated by miR-2188 over expression during early developmental stages, which is incompatible with normal ISV development in zebrafish. A decrease of *Nrp2a* expression is observed in 24 and 48 hpf embryos injected with the miR-2188 duplex, supporting the hypothesis that miR-2188 regulates this VEGF co-receptor. Conversely, knocking down miR-2188 with a MO_miR-2188_, results in *Nrp2a* up regulation at 24 and 48 hpf. Interestingly, at 48 hpf the increase in *Nrp2a* expression is observed between the somite boundaries and in the DA and PCV, regions where *Nrp2a* is barely expressed in normal conditions. This indicates that knocking down miR-2188 increases *Nrp2a* expression, which is likely responsible for the irregular ISV patterning that often results in DLAV defects. This phenotype is distinct from that obtained in embryos over expressing miR-2188, supporting the hypothesis that deregulation of the latter interferes with ISV patterning. Moreover, proper ISV patterning is rescued by injection of MO_miR-2188_ in embryos over expressing miR-2188.

Our data shows that miR-2188 interferes with proper ISV development by directly targeting *Nrp2a*, indicating that angiogenesis can be regulated at the level of VEGF receptors by miRNAs. One cannot exclude that this miRNA may also target other mRNAs involved in the VEGF pathway. Indeed, miRNAs can be master regulators of key pathways by targeting multiple mRNAs in a coordinated fashion [28], [33]–[35]. For example, miR-126 targets *Spred1* and *Pik3r2* genes, which are involved in the VEGF pathway showing that angiogenesis and vascular integrity can be disrupted by a single miRNA [28], [29]. Also, the neuronal enriched miR-128 regulates *Reelin* and *Dcx* and migratory potential of neuronal cells through distinct mechanisms [35], while miR-9 regulates the Fgf signaling by inhibiting *Fgf8*, *Fgfr1* and *Canopy1* and exerts a proneurogenesis effect by inhibiting expression of antineurogenic bHLH [33]. Therefore, one should not exclude the hypothesis that miR-2188 has multiple targets, which were not detected due to the high stringency of our initial prediction method. To clarify this issue, we have repeated the target prediction analysis for miR-2188 as before [15], by applying a lower stringent cutoff (with perfect seed match between nucleotides 2 and 7 and allowing up to 7 mismatches in the remaining sequence). This methodology was able to retrieve 8 additional putative targets for miR-2188, namely *Nfe2l1, Has2, Myst3, Cpla2, Atg13, Dnajc3, Dnaja3a and Pdik1l* (Table 2). Most of the predicted target genes are related with protein folding and heat shock response (*Atg13*, *Dnajc3* and *Dnaja3a*) and are necessary for the proper embryonic development (*Myst3*). Interestingly, *Cpla2* is involved in the VEGF signaling pathway and regulates endothelial cell cycle progression and angiogenesis [36]. In fact, it has been shown that this gene promotes the angiogenic tubule formation as its inhibition results in defects in the endothelial cell proliferation machinery [36]. Moreover, this gene is highly expressed in several carcinomas and is associated with tumor angiogenesis [37]. Due to the involvement of this putative target in angiogenesis, similarly to *Nrp2a*, future work should validate *Cpla2* as a miR-2188 target.

**Table 2:** Putative targets of dre-miR-2188.

Gene ID (ZFIN)	Gene name	GOMolecular Function	GOBiological process	GOCellular component
Nfe2l1	nuclear factor, erythroid derived2,-like 1a	Binding: DNA	Regulation of transcription	Nucleus
*Has2*	hyaluronan synthase 2	Hyaluronan synthaseactivity; Transferaseactivity, transferringhexosyl groups	atrioventricular valvedevelopment; cell migrationin hindbrain; cell migrationinvolved in gastrulation;dorsal convergence;embryonic heart tubedevelopment; heart looping;hyaluran biosyntheticprocess; mesodermal cellmigration; metabolic process;somitogenesis	Membrane
*Myst3*	K(lysine) acetyltransferase 6A	DNA binding; transferaseactivity; zinc ion binding	anterior/posterior patternspecification; cartilagedevelopment; embryonicbody morphogenesis;embryonic patternspecification; embryonicskeletal system development;embryonic viscerocraniummorphogenesis; histoneacetylation; neural crest cellfate specification;nucleosome assembly;regulation of transcription,DNA-dependent	Nucleus; Nuclear chromatin;Nucleosome;
*Cpla2*	cytosolic phospholipase a2	hydrolase activity;lysophospholipase activity;metal ion binding;phospholipase activity	lipid catabolic process;metabolic process; ovarianfollicle development;phospholipid catabolicprocess	cytoplasm; cytoplasmicmembrane-bounded vesicle;cytoplasmic vesicle
*Atg13*	Autophagy related protein 13	molecular	autophagy	cytoplasm; cytosol; pre-autophagosomal structure
*Dnajc3*	DnaJ (Hsp40) homolog, subfamily C,member 3	binding; heat shockprotein binding; unfoldedprotein binding;	protein folding	cellular component
*Dnaja3a*	DnaJ (Hsp40) homolog, subfamily A,member 3A	ATP binding; heat shockprotein binding; metalion binding; unfoldedprotein binding	protein folding; responseto heat	unknown
*Pdik1l*	PDLIM1 interacting kinase 1 like	ATP binding; kinaseactivity; nucleotidebinding; protein kinaseactivity; proteinserine/threonine kinaseactivity; transferaseactivity; transferaseactivity, transferringphosphorus-containinggroups	phosphorilation; proteinphosphorilation	nucleus

Nrp genes are widely conserved among vertebrate species. For example, human *Nrp2* and zebrafish *Nrp2a* orthologs have 57% similarity [19], but miR-2188 has not been identified to date in humans [38], suggesting that regulation of *Nrp2a* by miR-2188 may be zebrafish specific. This is in line with the hypothesis that recently evolved and non-conserved miRNAs may regulate species specific genes and may play a key role in evolutionary processes [39].

In conclusion, our data shows for the first time that miR-2188 targets *Nrp2a*, a positive regulator of angiogenesis, indicating that the VEGF pathway can be regulated by miRNAs controlling VEGF co-receptors.

## Materials and Methods

### Ethics Statement

All experiments were performed according to the European law for animal experiments (2010/63/EU). No specific ethics approval under EU guidelines was required for this project, as all zebrafish used in this study were between 0 and 5 days old. This is within the European law (Council Directive 86/609/EEC), which excludes foetal and embryonic forms.

Wild type AB and Tg(flk1-GFP)s843 zebrafish were maintained under the protocols approved by the Institutional Animal Ethics Committee of the University of Liège under application number 567. Zebrafish embryos were collected and kept at 28°C under standard laboratory conditions, and staged as described [40].

### miRNA Duplex Injections

A miR-2188 RNA duplex and a duplex containing a scrambled sequence were obtained as siRNAs from SIGMA:

miR-2188: sense 5′-AAGGUCCAACCUCACAUGUCC_RNA_-3′;

antisense 5′-ACAUGUGAGGUUGGACCUU_RNA_[dT][dT]-3′;

scrambled: sense 5′-GUGUAACACGUCUAUACGCCCA_RNA_-3′;

antisense 5′-GGCGUAUAGACGUGUUACAC_RNA_[dT][dT]-3′; and were injected into one-cell fertilized embryos at 2 µM. Approximately 1000 pL of 2 µM (miR-2188 and scrambled duplex) diluted in TE buffer and 5% Rhodamine were injected in one cell embryos. Embryos were staged and examined for phenotypic alterations.

### Morpholino Injections

The miR-2188 morpholino (MO) was obtained from Gene Tools, diluted in Daniaeu Buffer and 5% Rhodamine and 1000 pL were injected in one cell embryos at 8 ng/µL. 1000 pL of a control MO from Gene Tools was also injected at 8 ng/µl. Phenotypes were assessed at 24 hpf and after blood circulation was established.

### Image Aquisition

24 hpf and 48 hpf control and injected (either with miRNA duplexes or MOs) Tg(flk1-GFP)s843 embryos were dechorionated and fixed in 4% PFA at 4°C for 4 h, washed 3 times in PBS-T and stored at 4°C. For imaging, stored embryos were mounted in Prolong and visualized using an OLYMPUS FV1000 confocal microscope, equipped with FV-10ASW software, FITC filters and a 488 nm laser. Images from control and injected embryos with the duplexes or the MOs were acquired with 20x, 40x and 60x objectives.

### Quantitative Real-time PCR

Total RNA was extracted from embryos injected with the miRNA duplexes, from embryos injected with miRNA MOs and from control embryos at 48 hpf using Qiazol (Qiagen), following the manufacturer’s protocol. Samples were treated with DNase to remove any contaminating genomic DNA. RNA quantity and quality were assessed using the Nanodrop and Agilent 2100 bioanalyzer systems, respectively. Samples with a RNA integrity number (RIN) above 7 were used in this study.

Quantification of miR-2188 expression was carried out using NCode™ miRNA first-strand cDNA module and Platinum® SYBR® Green qPCR Super Mix-UDG from Invitrogen. Briefly, miRNAs were polyadenylated using poly-A polymerase and ATP. cDNA was then prepared from 500 ng of total RNA with the SuperScript® III RT and a Universal RT primer, following the manufacturer’s protocol. Quantitative real-time PCR (qPCR) was performed with the synthesized cDNA using SYBR® Green detection reagent, the Universal qPCR primer provided in the kit, and a forward primer designed to target miR-2188 (5′-AAGGTCCAACCTCACATGTCC-3′). U6 small RNA was used as an endogenous control for normalizing miRNA levels.

Quantification of *Nrp2a* was carried out using Power SYBR® Green PCR Master Mix from Applied Biosystems. cDNA was prepared from 500 ng of total RNA with the SuperScript®II RT (Invitrogen) following the manufacturer’s instructions. qPCR was performed with the synthesized cDNA using Power SYBR® Green detection reagent and forward (5′-TGGAGCTACTCTGTTCCAGC-3′) and reverse (5′- TGTCCAAAGAGAGACTGGGA-3′) *Nrp2a* primers. Tubulin α-1 was used as an internal control for normalizing mRNA levels as its expression level is relatively constant across tissues and it is not significantly altered by experimental conditions [41].

Efficiency of primers was determined after the generation of a standard curve. All reverse transcriptions and no-template controls were carried out simultaneously following the RT step. qPCR was carried out using the ABI Prism 7500 Sequence Detector System (Applied Biosystems). Reactions were incubated in 96-well optical plates and cycling began with template denaturation at 95°C for 2 min, followed by 50 cycles at 95°C for 15 seconds and 60°C for 35 seconds for miR-2188 detection. For *Nrp2a* detection, cycling began with template denaturation at 95°C for 10 min, followed by 40 cycles at 95°C for 15 seconds and 60°C for 1 min. The threshold cycle data (CT) and baselines were determined using auto settings. All assays including no template controls were carried out in triplicate. The REST software [42] was used to quantify Nrp2a and miRNA levels. In our dataset the control samples were the scrambled (for duplex injection) or MO_ctrl_ (for MO injections).

### In Situ Hybridization

*In situ* hybridization was carried out as described [43] with a *Nrp2a* probe kindly provided by the Laboratory of Molecular Biology and Genetic Engineering, Liège University. After staining, embryos were washed 4 times with TBS-T. Embryos were mounted in 100% glycerol and photographed using a color digital camera.

miR-2188 ISH was carried out with a LNA probe from Exiqon (/5DigN/GGACATGTGAGGTTGGACCTT/3Dig_N/, Tm 79°C). Briefly, 24 hpf and 48 hpf embryos were collected and prepared for ISH as described above. Incubations with hybridization mix and miR-2188 probe were performed at 61°C to minimize unspecific hybridization. 10 nM of miR-2188 probe were used for the ISH.

### Sensor assay

The pCS2+ plasmid containing eGFP (gift from Dr. Hyangshuk Rhim, Research Institute of Molecular genetics, Catholic University of Korea), was used in this study. The 3′UTR of Nrp2a containing putative miR-2188 binding sites was fused to the 3′ end of eGFP using StuI and XhoI. The 3′ UTR of Nrp2a was amplified using the following primers:

Forward: 5′-GCGCAGGCCTTCCTAAAATGACCTCAAAGT-3′.

Reverse: 5′-GCGCCTCGAGGTATATCGAGTGTTTAAGAC-3′.

Nrp2a 3′UTR sequences carrying point mutations in the predicted miR-2188 binding sites were engineered by site directed mutagenesis. A M1 construct containing mutations in the binding site-1 (BS-1) was generated using the following primers: Forward: 5′-GGGAGATCTGTGGC**C**GG**GA**C**CC**CGAGCCATGAACTTCTGTCC-3′ Reverse: 5′-GGACAGAAGTTCATGGCTCGGGGTCCCGGCCACAGATGTCCC-3′.

A M2 construct containing the mutation in the binding site-2 (BS-2) was generated with the following primers:

Forward: 5′-TTTGCAAACGGGTTAG**TG**C**TG**AACACTGAGCCTTGAGG-3′.

Reverse: 5'-CCTCAAGGCTCAGTGTTCAGCACTAACCCGTTTGCAAA-3'.

Highlighted nucleotides show the mutations inserted in the 3′UTR. Point mutations were PCR amplified and fused to the 3′ end of eGFP, as described above. For all constructs, capped mRNA was prepared to avoid degradation after microinjection, using the mMESSAGE mMACHINE SP6 Kit (Ambion), according to manufacturer’s protocol. The mRNA (final concentration 50 ng/µL) was injected with either miR-2188 duplex or the control miRNA duplex containing a scrambled miRNA sequence (final concentration 2 µM). Approximately 1000 pL of the mixture of mRNA and duplex were injected and embryos were pooled and prepared for western blot analysis at 24 hpf.

### Western Blot

24 hpf embryos were pooled (20–30 embryos each) and embryo extracts were prepared after dechorionation and deyolking. 2xSDS sample buffer was added, followed by denaturation at 95°C for 5 min followed by vortexing. The protein solution was centrifuged for 2 min using a table-top centrifuge and samples were directly loaded onto 15% PAA protein gels and electrophoresed in SDS running buffer. Proteins were transferred overnight at 4°C to PVDF Hybond-P membranes (GE Healthcare), which were blocked for 1 h with 4% nonfat dry milk in TBS-T. Immunodetection was carried out using monoclonal anti-Green Fluorescent Protein mouse antibody (1∶1000) from Clonetech and anti β-tubulin mouse antibody (1∶500) from Invitrogen, as primary antibodies. IRDye®800 CW Goat anti-mouse IgG from LI-COR® was used as secondary antibody (1∶10000). Detection was carried out using Odissey® Imaging System.

## Supporting Information

Figure S1 **Developmental defects observed after miR-2188 duplex and Mo_miR-2188_ injections.** At 48 hpf developmental defects were quantified. Scrambled and MO_Ctrl_ injected embryos developed similarly to non-injected embryos, while embryos injected with the miR-2188 duplex revealed high incidence of pericardial edema (70%) and circulation arrest (50%). More than 50% of the embryos lacked one or more ISVs. MO_miR-2188_ injected embryos showed ISV branching (50%) that often resulted in dorsal longitudinal anastomotic vessel (DLAV) defects (50%). Twenty four embryos were analyzed per condition (n>3).
(TIFF)

Figure S2 **ISV patterning is rescued after injection of Mo_miR-2188_ in embryos overexpressing miR-2188.** Visualization of 48 hpf embryo blood vessels using confocal microscopy (20x). ISVs of miR-2188 injected embryos were thinner and displayed ISV patterning defects, when compared to non-injected embryos. Injection of MO_ miR-2188_ in embryos over expression miR-2188 rescued the ISV patterning.
(TIFF)
