# Supplementary figures and images for: Dre-miR-2188 Targets Nrp2a and Mediates Proper Intersegmental Vessel Development in Zebrafish Embryos
Source: PLoS One. 2012 Jun 22;7(6):e39417. doi: 10.1371/journal.pone.0039417 (PMC3382224; doi:10.1371/journal.pone.0039417)

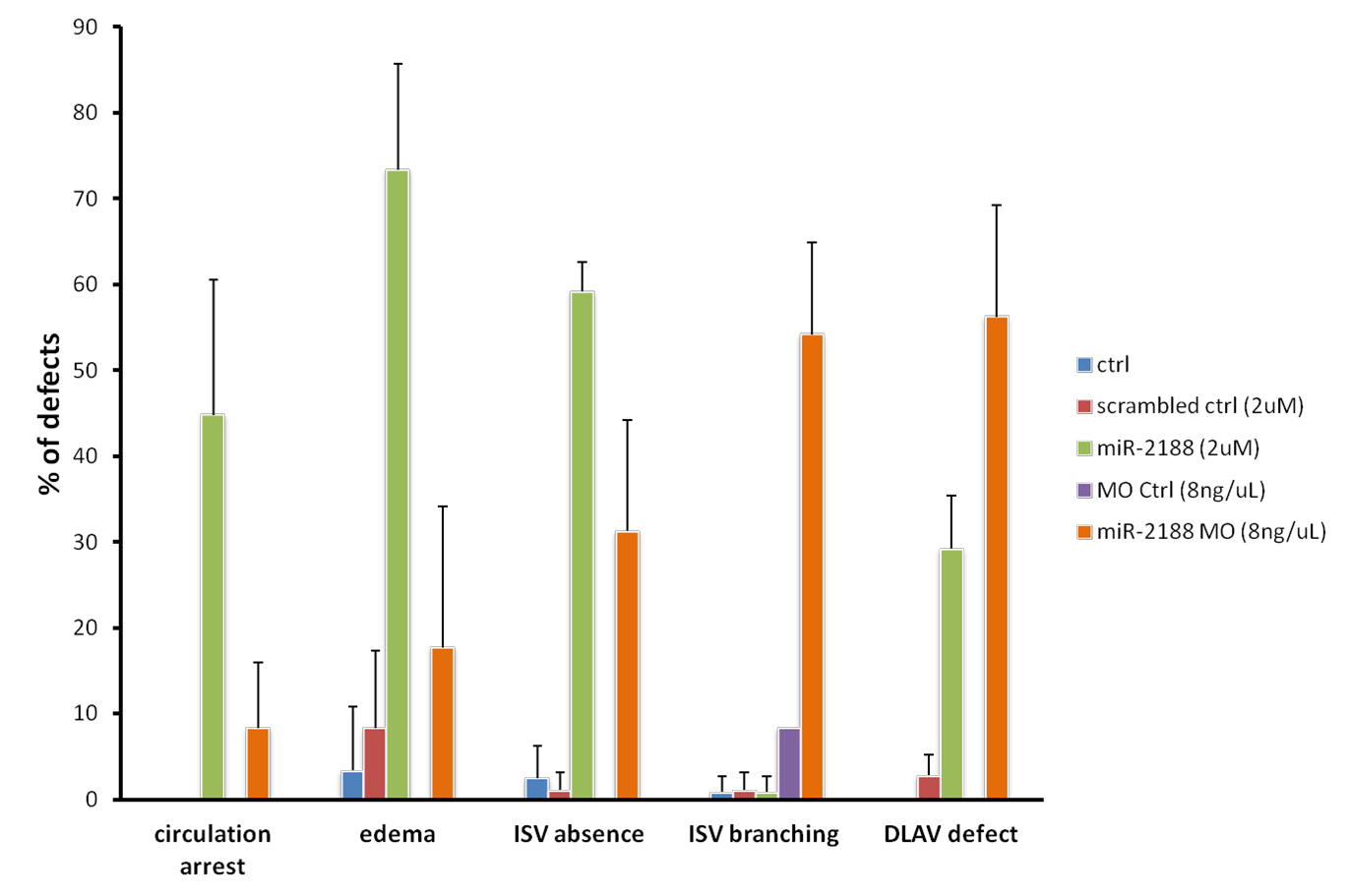

Supplement: Figure S1 — Developmental defects observed after miR-2188 duplex and MomiR-2188 injections. At 48 hpf developmental defects were quantified. Scrambled and MOCtrl injected embryos developed similarly to non-injected embryos, while embryos injected with the miR-2188 duplex revealed high incidence of pericardial edema (70%) and circulation arrest (50%). More than 50% of the embryos lacked one or more ISVs. MOmiR-2188 injected embryos showed ISV branching (50%) that often resulted in dorsal longitudinal anastomotic vessel (DLAV) defects (50%). Twenty four embryos were analyzed per condition (n>3). (TIFF) [file pone.0039417.s001.tiff]

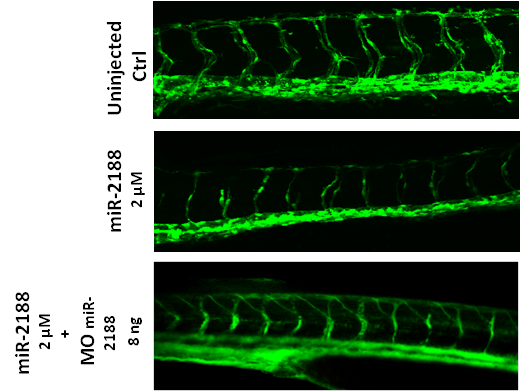

Supplement: Figure S2 — ISV patterning is rescued after injection of MomiR-2188 in embryos overexpressing miR-2188. Visualization of 48 hpf embryo blood vessels using confocal microscopy (20x). ISVs of miR-2188 injected embryos were thinner and displayed ISV patterning defects, when compared to non-injected embryos. Injection of MO miR-2188 in embryos over expression miR-2188 rescued the ISV patterning. (TIFF) [file pone.0039417.s002.tiff]
